# Returners and explorers dichotomy in the face of natural hazards

**DOI:** 10.1038/s41598-024-64087-4

**Published:** 2024-06-08

**Authors:** Zeyu He, Yujie Hu, Leo L. Duan, George Michailidis

**Affiliations:** 1 Department of Geography, University of Florida, Gainesville, FL 32611 USA; 2 Department of Statistics, University of Florida, Gainesville, FL 32611 USA; 3 Department of Statistics and Data Science, University of California Los Angeles, Los Angeles, CA 90095 USA

**Keywords:** Natural hazards, Environmental social sciences, Sustainability

## Abstract

Understanding human mobility patterns amid natural hazards is crucial for enhancing urban emergency responses and rescue operations. Existing research on human mobility has delineated two primary types of individuals: returners, who exhibit a tendency to frequent a limited number of locations, and explorers, characterized by a more diverse range of movement across various places. Yet, whether this mobility dichotomy endures in the context of natural hazards remains underexplored. This study addresses this gap by examining anonymized high-resolution mobile phone location data from Lee County, Florida residents, aiming to unravel the dynamics of these distinct mobility groups throughout different phases of Hurricane Ian. The results indicate that returners and explorers maintained their distinct mobility characteristics even during the hurricane, showing increased separability. Before the hurricane, returners favored shorter trips, while explorers embarked on longer journeys, a trend that continued during the hurricane. However, the hurricane heightened people’s inclination to explore, leading to a notable increase in longer-distance travel for both groups, likely influenced by evacuation considerations. Spatially, both groups exhibited an uptick in trips towards the southern regions, away from the hurricane’s path, particularly converging on major destinations such as Miami, Fort Lauderdale, Naples, and West Palm Beach during the hurricane.

## Introduction

The incidence of natural disasters is escalating, driven by growing populations, rapid urbanization, and changing climate dynamics^1–5^. The Centre for Research on the Epidemiology of Disasters^6^ reports 432 global incidents in 2021 alone, which resulted in 10,492 deaths, impacted over 101.8 million people, and led to economic damages approaching 252 billion USD. Corroborating this trend, the United Nations Office for Disaster Risk Reduction’s 2022 report forecasts a continued increase in these adversities^7^. Given the potential for profound disruptions in human movement, it is imperative for policymakers and urban planners to rethink strategies and devise responsive approaches. This emerging challenge is gaining significant academic focus^8–14^.

In the past decade, massive datasets of location traces emerged, allowing characterization of human movement patterns at an unprecedented scale. Examples include digital traces produced by: (1) GPS devices embedded in vehicles^15,16^, bike sharing systems^17^, and smartphones^18,19^; (2) cellular networks that record call detail records (CDR) about mobile subscribers’ communication activities^20,21^; and (3) geotagged posts from social media platform^22,23^. It is thus not surprising that a vast scientific production on human mobility theories and modeling has since emerged^24^.

Existing research in human mobility, for instance, has pinpointed some consistent statistical patterns that characterize human movements^25^. Primarily, human travels often exhibit a power-law distribution^20,26^, though other distributions like exponential and log-normal are also observed^27^. Moreover, human movements are typically predictable and often cycle between a few frequently visited locations, captured by Zipf’s law in visitation frequency^21^. Interestingly, two primary mobility mechanisms emerge: exploration and preferential return. These mechanisms divide individuals into two distinct mobility classes: returners and explorers^16^. Returners consistently frequent a limited set of familiar locations, such as homes or workplaces, displaying a strong periodic or routine pattern. Explorers, in contrast, venture into new and varied locations. They exhibit a higher propensity to explore unfamiliar places they haven’t visited frequently, making their movements less predictable than those of returners.

However, it remains unclear if the returners and explorers dichotomy persists in disaster situations. This is because that the mobility dichotomy emerged under assumptions of stable social conditions, without considering potential disruptions from significant external events like natural hazards^28–30^. Notably, many studies spanning geography, transportation, and other social sciences have documented pronounced shifts in travel behaviors under hazardous conditions^14,30–33^, which might alter the established dichotomy. For example, Hurricanes Matthew and Harvey led to a considerable reduction in both the distance people traveled and the area they covered^34^. A similar impact was observed during the severe winter storms of 2015 in the United States, where individuals exhibited disruptions in displacement and radius of gyration. This was characterized by an increase in short-distance trips and a decrease in long-distance trips^35^. This pattern aligns with the findings during Hurricane Sandy^36^, Hurricane Dorian^37^, and Typhoon Mangkhut^38^. This pattern was also observed in the aftermath of the 2017 Jiuzhaigou earthquake^39^. However, alternative viewpoints on these disruptions have been presented in the literature. For example, following the 2010 Haiti earthquake, there was an observed expansion in individuals’ daily travel distances^40^. In contrast to hurricanes and storms, a significant increase in the number of trips people took after an earthquake was observed^29^. Beyond this, communities with different socio-economic conditions have also shown different evacuation responses to disasters^41^. But they have yet to explore the possible impacts to the recognized returners and explorers dichotomy and their movement patterns during these events. Furthermore, the mobility dichotomy and other statistical patterns have been primarily derived from long-term mobility data, often spanning months^16,20^, thereby overlooking short-term mobility fluctuations. This limitation arises from the coarse spatiotemporal resolution inherent in prevalent datasets like mobile phone CDRs and geotagged Twitter entries^30,34,42^, necessitating aggregation to broader spatial and temporal scales to enhance data robustness^22,43^.

Utilizing high-resolution anonymized mobile phone location data from residents of Lee County, Florida, this study aims to examine the presence of returners and explorers and their corresponding movement patterns throughout Hurricane Ian in 2022. It further conducts a spatial analysis, visualizing the movement patterns of these two mobility groups. This research stands out for several reasons. Primarily, it is the first attempt to investigate the generalizability of the returners and explorers dichotomy in the context of natural disasters. Additionally, the detailed spatial exploration enriches our understanding of mobility trends, filling gaps left by prior studies centered on universal statistical patterns. Lastly, it offers insights into the largely unexplored impacts of Hurricane Ian, which inflicted significant damage upon Florida residents.

## Results

**Figure 1:**
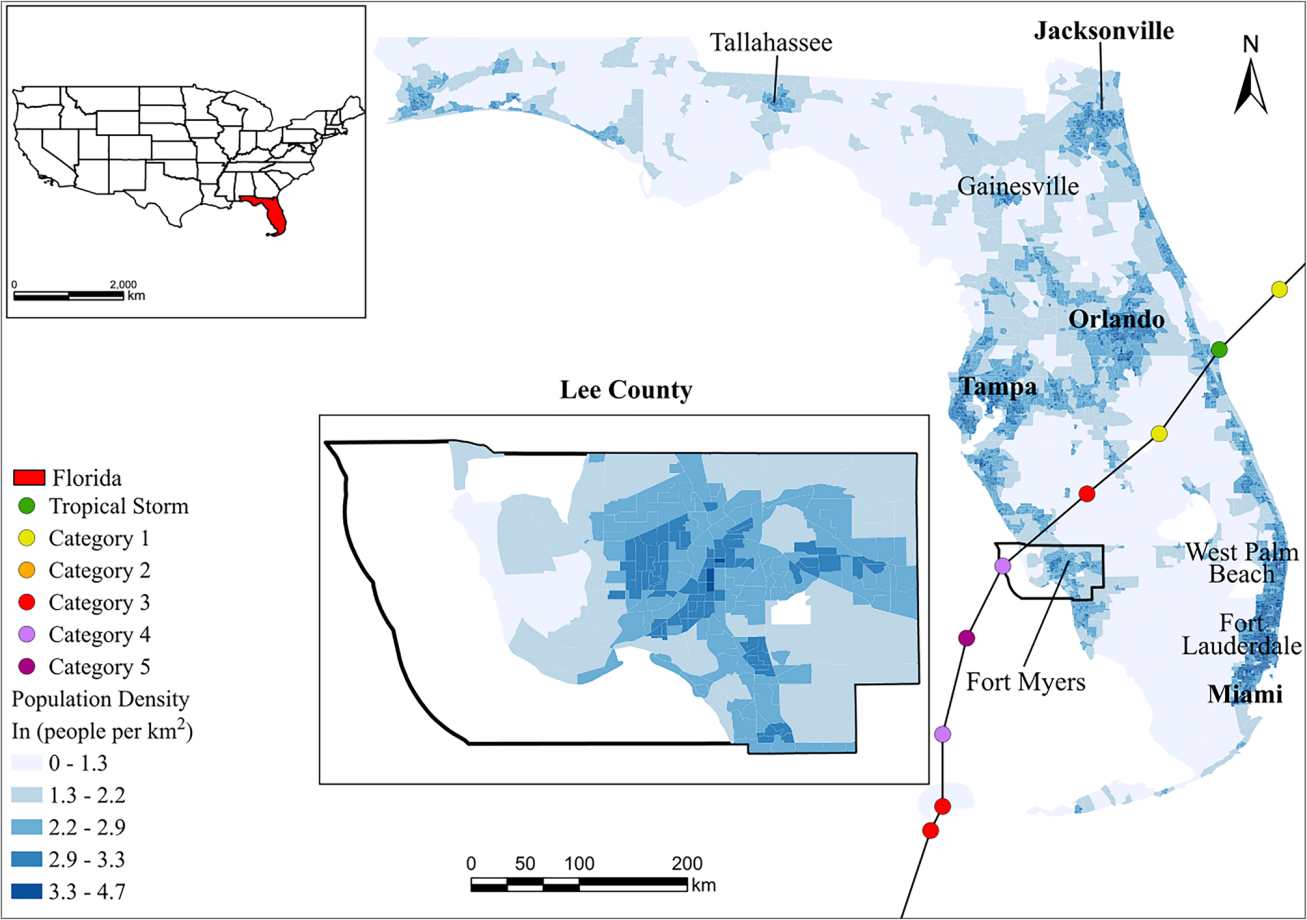
The study area and the track of Hurricane Ian [Figure created using ArcGIS Pro 3.1.3: https://pro.arcgis.com].

This study delves into the mobility patterns of residents in Lee County, Florida, during the catastrophic Hurricane Ian. Striking as a category 4 hurricane, Ian made landfall on Cayo Costa Island in southwest Florida on September 28, 2022, at 19:05 UTC. It traversed northeastward across the state between September 28 and 29, diminishing to a tropical storm over eastern Florida^44^. The hurricane resulted in the loss of 105 lives and left over two million households without power, making it the most damaging hurricane in the state’s history. Lee County experienced the most devastating effects of the storm, particularly in Fort Myers Beach, Sanibel Island, and Bonita Springs. Ian’s forceful 10–15 ft (3.0–4.6 m) storm surge inundated these areas, which are situated just south of the hurricane’s landfall point^45^. Figure 1 illustrates the study area and the path of the hurricane.

To gain a comprehensive understanding of Hurricane Ian’s influence on Florida’s mobility patterns, this study specifically focuses on movements from September 27 to 30, correlating with the storm’s timeline and the corresponding evacuation mandates. To contextualize these findings, mobility on four consecutive days both two weeks before and after the hurricane is also examined, serving as a standard reference. This methodology offers a detailed comparison between hurricane-influenced mobility and routine movement patterns. Our focus was narrowed to movements within Florida, specifically involving Lee County residents, ensuring the relevance of data to the hurricane’s ramifications. To achieve this, the study extracts and analyzes mobile phone location data from 304,248 anonymous device users who visited Lee County at least once between August 1, 2022, and October 31, 2022. For each device user, their primary Census Block Group of residence is determined through three metrics: visit frequency to a location in the preceding month, the average time spent there daily, and the duration at that location during nighttime hours. Table 1 presents an overview of the refined dataset. Supplementary Fig. 1 displays the strong representativeness of the samples across the three study periods.

**Table 1:** An overview of the refined dataset.

Study period	Date range	Number of devices	Number of stops	Average positioning accuracy (m)
Pre-hurricane	09/13/2022–09/16/2022	33,401	795,316	15
Hurricane	09/27/2022–09/30/2022	13,222	218,163	15
Post-hurricane	10/11/2022–10/14/2022	28,643	666,276	15

### Mobility trends across hurricane phases for general users

Supplementary Fig. 2 visualizes the fitting results for both displacement (*D*) and radius of gyration (*r*_*g*_) across the three stages, with Supplementary Table 1 detailing the maximum likelihood estimates of these fits. From Supplementary Table 1, it’s evident that the truncated power law distribution provides the most optimal fit for both measures across all periods. During the hurricane, *λ* values are notably higher compared to before and after the event, while *α* values show the reverse trend.

This study further probes into a daily-scale analysis using the truncated power law to fit both the displacement and radius of gyration. Supplementary Fig. 3 visualizes these results. Notably, the truncated power law consistently delivers the best fit. The trend of lower *α* values and higher *λ* values throughout the three hurricane stages remains consistent. These findings underscore the robustness of human movement patterns, demonstrating that even amid this major hurricane, the displacement and activity range persistently followed a truncated power-law distribution. This observation aligns with several studies that identified a power-law distribution in human displacements during natural hazards^28^.

Furthermore, this study investigates the temporal evolution of the spatial distribution of activity stops among Lee County residents across three distinct periods. The results are presented in Supplementary Fig. 4. A noticeable decrease in activity in the northern part of Lee County and a significant drop near the Fred C. Babcock/Cecil M. Webb Wildlife Management Area and Disney World during the hurricane are revealed, consistent with the predicted path of the hurricane moving northward. Conversely, activity in the south, specifically in areas like the Everglades and Francis S. Taylor Wildlife Management Area, Fort Lauderdale-Hollywood International Airport, and Miami International Airport, markedly increased. This suggests that individuals might seek open outdoor areas to avoid the hurricane or opt for air travel as a means of evacuation. In the post-hurricane period, activity levels reverted to their previous state.

### Classification of returners and explorers across hurricane phases

**Figure 2:**
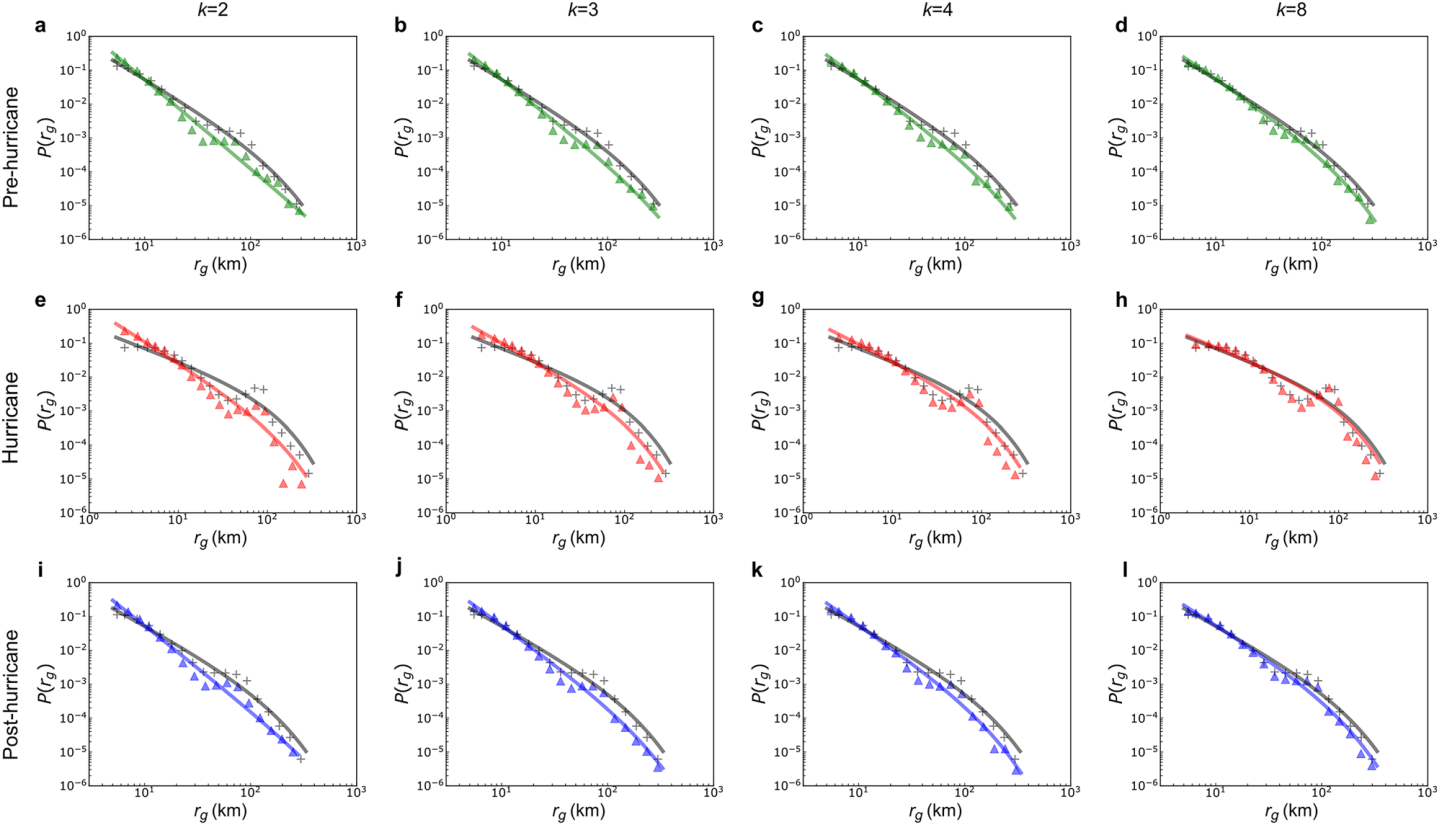
The distributions of $r_{g}$ and $r_{g}^{( k )}$, with $k$ = 2, 3, 4, 8, in the pre-hurricane (**a**–**d**), hurricane (**e**–**h**), and post-hurricane (**i**–**l**) periods. Green, red, and blue triangles represent the empirical data of $r_{g}^{( k )}$ for different values of $k$ before, during, and after the hurricane, respectively. Green, red, and blue solid lines correspond to the fitted truncated power-law lines for these periods. Black crosses represent the empirical data of $r_{g}$, and the black solid line represents the fitted truncated power-law line.

To differentiate between returners and explorers, this study employs the *k*-radius of gyration^16^. To address potential variations in $r_{g}$ due to the choice of the top *k* frequently visited locations, this study evaluates $r_{g}^{(k)}$ using four specific values of *k*: 2, 3, 4, and 8. The selection of 2 takes into account frequent visits to home and workplace for most individuals, while choosing 8, representing the upper limit of visited locations, encompasses nearly everyone in the data. By considering 2, 3, 4, and 8, this analysis comprehensively captures the primary trends in *r*_*g*_. Note that results for *k* = 5, 6, and 7 exhibit a consistent trend between *k* = 4 and *k* = 8 and are therefore omitted. The results are displayed in Fig. 2, and detailed fitting parameters are presented in Supplementary Table 2. A relatively significant disparity was observed between the *r*_*g*_ and $r_{g}^{(k)}$ when *k* = 2, indicating that *r*_*g*_ of a subset of individuals cannot be adequately characterized by merely two frequently visited locations. This phenomenon becomes more pronounced under hurricane conditions. As the value of *k* gradually increases, the observed discrepancy diminishes, suggesting that the number of individuals whose *r*_*g*_ is dominated by *k* frequently visited locations is increasing. By the time when *k* reaches 8, the disparity becomes minimal, demonstrating that eight frequently visited locations are sufficient to represent *r*_*g*_ of the majority of individuals. It is evident that, across all three periods, the $\alpha$ value for $r_{g}^{(k)}$ consistently surpasses that of *r*_*g*_, diminishing as *k* rises. The $\lambda$ value, on the other hand, presents a more complex trend: only for $r_{g}^{(2)}$ does the $\lambda$ value fall below that of $r_{g}$, whereas it surpasses that of $r_{g}$ in other instances. Notably, the onset of the hurricane sees a marked drop in the $\alpha$ value, with $\lambda$ exhibiting the opposite trend. Furthermore, as *k* increases, the fitted curve for $r_{g}^{(k)}$ converges towards that of $r_{g}$, suggesting an inherent upper bound for frequent visits by individuals. Another noteworthy observation emerges around the 70 km mark in the probability distribution of $r_{g}$. Before and after the hurricane, this value stays consistent, yet surges during the hurricane itself.

**Figure 3:**
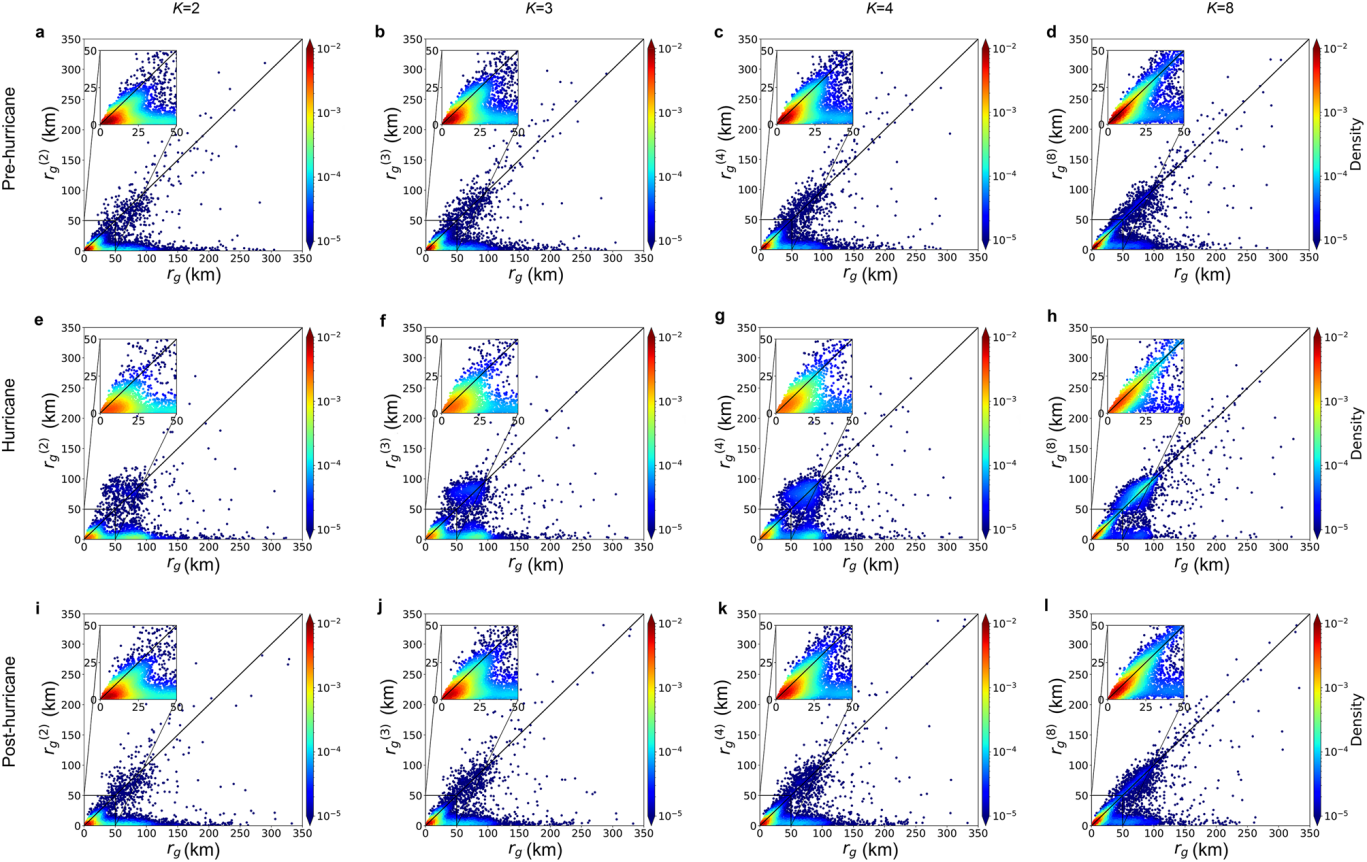
The scatter plots represent the relationship between $r_{g}^{( k )}$ and $r_{g}$ for $k$ = 2, 3, 4, 8 in the pre-hurricane (**a**–**d**), hurricane (**e**–**h**), and post-hurricane (**i**–**l**) periods. Each point is colored from blue to red, indicating the density of points in the corresponding region. The insets magnify the origin of the plot to [0, 50 km] and the solid diagonal line indicates where $r_{g}^{( k )}$ equals $r_{g}$.

Figure 3 presents the density analysis contrasting $r_{g}^{( k )}$ and $r_{g}$, unveiling pronounced variations between the two mobility groups across different *k* values. For the three periods analyzed, data points mainly cluster around the diagonal and horizontal axes. This suggests that for one group, the characteristic travel distance aligned closely with their top *k* frequented locations, hinting at a *k*-returner behavior. Conversely, the other group, termed *k*-explorers, showed movement patterns less aligned with their top *k* locations. A noteworthy observation during the hurricane period is the concentration of $r_{g}$ and $r_{g}^{( k )}$ around 70 km, an increase in the travel range of individuals. As *k* ascends, the clustering grows more diagonally, indicating a shift from *k*-explorer to *k*-returner behaviors—especially pronounced amidst the hurricane.

**Figure 4:**
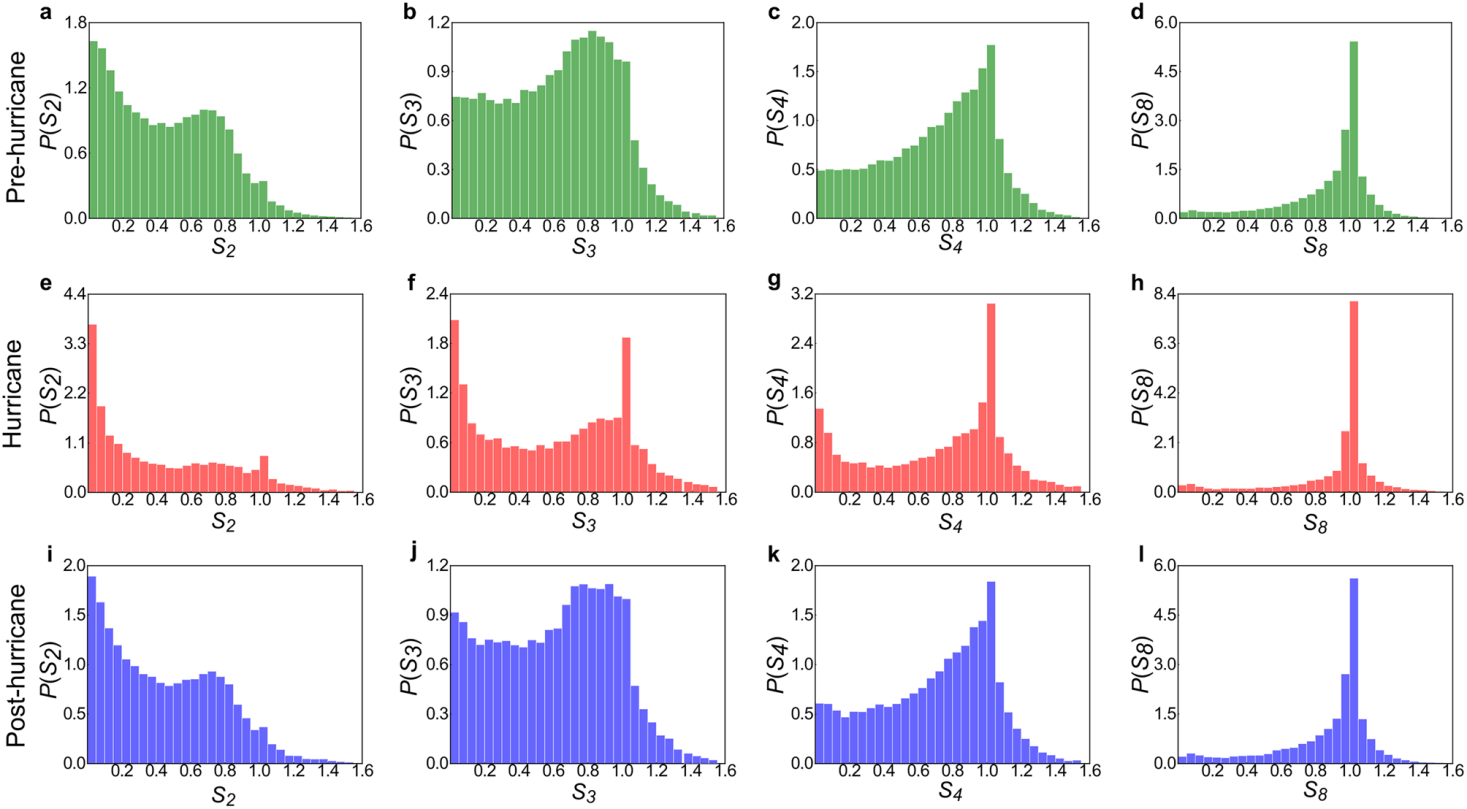
The distribution $P( {S_{k} } )$ of the ratio $S_{k} = r_{g}^{( k )} /r_{g}$ measured in the pre-hurricane (**a**–**d**), hurricane (**e**–**h**), and post-hurricane (**i**–**l**) periods. The peak at $S_{k}$ = 0 corresponds to explorers, while the $S_{k}$ = 1 peak corresponds to returners.

To provide a more detailed illustration of how different values of *k* impact the identification of the two mobility groups, Fig. 4 highlights the relationship between $r_{g}^{( k )}$ and $r_{g}$ using the metric $S_{k} = r_{g}^{( k )} /r_{g}$. Peaks at $S_{k} = 0$ and $S_{k} = 1$ represent *k*-explorers and *k*-returners, respectively. Initially, when *k* is set to 2, the probability density peaks at $S_{k} = 0$ for all three periods. This suggests that characterizing the daily activities of Lee County residents in terms of only two frequently visited locations is challenging. However, there is a noticeable small peak at $S_{k} = 1$ during the time of the hurricane compared to the pre-hurricane period. This indicates a tendency to limit the number of places people visit, which becomes more pronounced at $k = 3$. At this point, the probability densities for all three periods sharply decrease at $S_{k} = 0$ and increase at $S_{k} = 1$. An unusually pronounced peak at both locations, especially during the hurricane stage, reveals the presence of these two groups in hurricane conditions more so than in normal conditions. As *k* increases to 4, the peaks for all three periods are centered at $S_{k} = 1$, revealing that the vast majority of Lee County residents are limited to four frequently visited locations. This trend continues as *k* grows, with the probability density at $S_{k} = 1$ showing a clear upward trend. Notably, as *k* reaches 8, there is an apparent peak at $S_{k} = 1$, suggesting an upper limit on the number of daily visit locations.

While the analysis of $r_{g}^{( k )}$ and $r_{g}$ has illuminated the presence of both groups over time and the shifts accompanying different *k* values, it is essential to quantify these changes for a more nuanced understanding. To achieve this, Lee County residents are categorized based on mobile phone data from each of the three time periods, and their share of the total population is calculated and compared. The results, presented in Supplementary Fig. 5, indicate that the general trend of the share of returners (explorers) increasing (decreasing) with an increasing value of *k* remains consistent across the three periods. Notably, at *k* = 2, the percentage of explorers markedly surpasses that of returners, with the most substantial gap observed during the hurricane period. Throughout the three stages, this pattern reverses beginning at *k* = 3, with a considerably higher percentage of returners compared to explorers, and the disparity expands with increasing *k*.

### Mobility trends comparison between returners and explorers across hurricane phases

**Figure 5:**
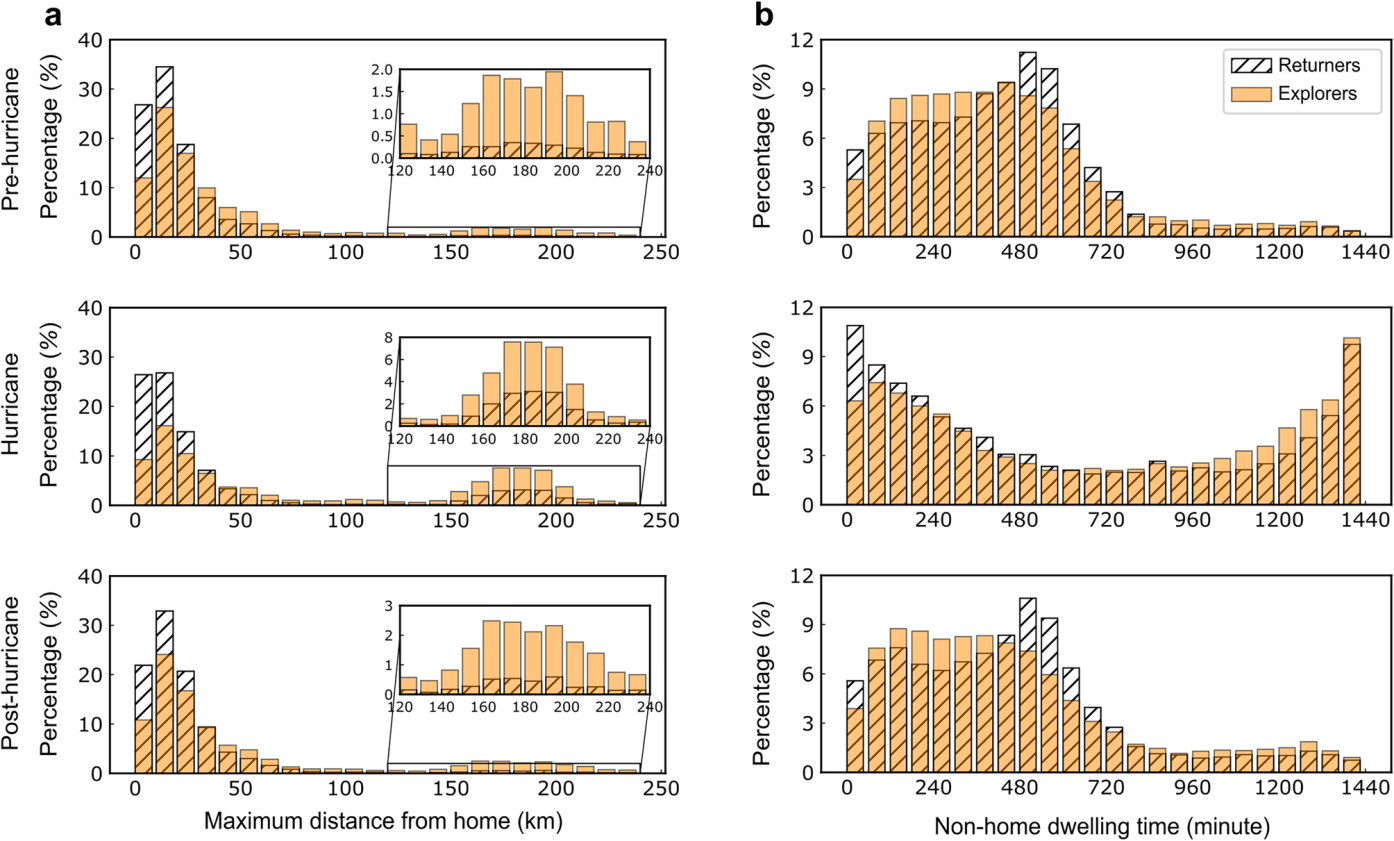
Maximum distance from home with an inset zoom on [120 km, 240 km] (**a**) and non-home dwelling time (**b**) for two groups across three periods.

In addition to examining changes in the proportion of the two mobility groups at different *k* values during the three periods, this study also analyzes mobility patterns of the two groups. This analysis specifically investigates alterations in their maximum travel distance from home and the duration of time spent away from home, employing *k* = 3. The selection of *k* = 3 is based on the emergence of dual peaks of *P*(*S*_*k*_), particularly noticeable during the hurricane stage at *S*_*k*_ = 0 and *S*_*k*_ = 1 in Fig. 4. Additionally, there is a clear shift in the relative distribution of the proportion of returners and explorers at *k* = 3 in Supplementary Fig. 5. Figure 5a illustrates the distribution of the maximum distance from home for both groups across the three periods, with the vertical axis representing their proportion of the total population. Supplementary Table 3 shows that the distributions are significantly different between the two groups. Remarkably, during the hurricane stage, both groups experienced a substantial increase in the share of trips within 160–200 km. Interestingly, in the pre-hurricane stage, returners demonstrated a larger share of short-distance trips compared to explorers, while explorers tended to favor longer distance trips. However, the hurricane evidently influenced these patterns, narrowing the gap in the maximum distance traveled between the two groups.

Furthermore, both groups displayed variations in the duration of time spent away from home, as depicted in Fig. 5b. While Supplementary Table 3 shows that the distributions between these two groups are not significantly different, it is still possible to see some of the subtle variations and effects of the hurricane on their movement patterns. It’s worth noting that a 0-min non-home dwelling time indicates a full stay at home. Under non-hurricane conditions, returners, in comparison to explorers, are more inclined to either stay at home (0-min non-home dwelling time) or venture outside home for 480–720 min (8–12 h), while explorers, relative to returners, are more likely to stay outside for 60–480 min (1–8 h). This distinct mobility pattern, however, dissipates during the hurricane. Specifically, both groups exhibited a shared bi-modal distribution pattern with the first mode (more prominent) at 0 min and the second mode at 1440 min (24 h). In other words, the stay time patterns of both returners and explorers can be distilled into two types—staying home or venturing outside for 23–24 h—possibly corresponding to non-evacuees and evacuees, respectively.

**Figure 6:**
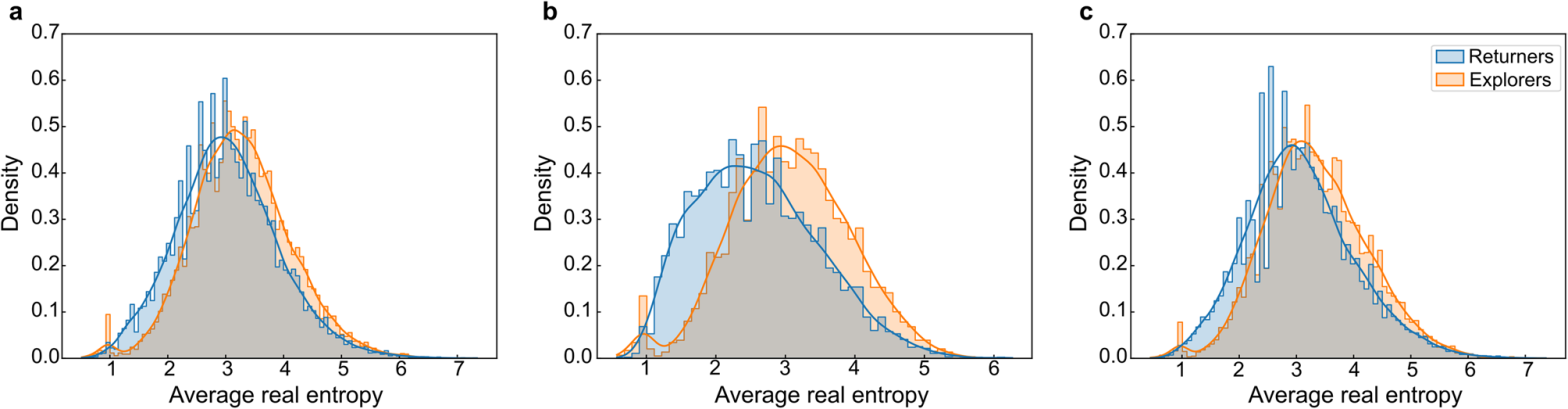
Density distribution of average real entropy for returners and explorers in the pre-hurricane (**a**), hurricane (**b**), and post-hurricane (**c**) periods. The orange line represents the kernel density estimate (KDE) curve for explorers, while the blue line represents the KDE curve for returners.

Moreover, to gain deeper insights into the disparities between the two groups, this study also analyzes the per capita real entropy across three distinct periods, as well as their dynamic transformations in response to hurricane impacts. The results shown in Fig. 6 exhibit discrepancies between the groups across these timeframes, with Supplementary Table 4 confirming the statistical significance of these distinctions. It is evident that, across all three periods, explorers demonstrate a higher per capita real entropy compared to returners, particularly during the hurricane, where this contrast becomes more pronounced. The movement patterns of explorers are characterized by greater randomness and unpredictability, while those of returners are more conservative and stable. This difference is further corroborated by the dynamic transitions observed post hurricane impact. Supplementary Fig. 6 illustrates that following the hurricane, 37.1% of returners transitioned into explorers, while 47.36% of explorers shifted to returners. This suggests that the more predictable movement patterns of returners likely render them less susceptible to the disruptions caused by the hurricane.

For both groups, while the number of long-distance trips increased during the hurricane, as shown in Fig. 5a, the total number of trips significantly decreased, reaching its lowest point on September 28, highlighting the hurricane’s profound impact on mobility behavior. Notably, Supplementary Fig. 7 also reveals a peak in the number of trips taken on Fridays and a trough on Sundays before the hurricane. Moreover, it is worth mentioning that just two weeks after the hurricane, people’s travel behavior in terms of the number of trips had nearly returned to pre-hurricane levels.

### Spatiotemporal mobility patterns comparison between returners and explorers across hurricane phases

Next, this study examines spatiotemporal mobility patterns. As shown in Supplementary Fig. 8, the percentage of individuals traveling remains consistently higher (significant at the 0.01 level—see Supplementary Table 3) among explorers than returners from September 27th to September 30th, regardless of the chosen value of *k*. This suggests a greater inclination among explorers to venture out from their homes during the hurricane’s impact.

**Figure 7:**
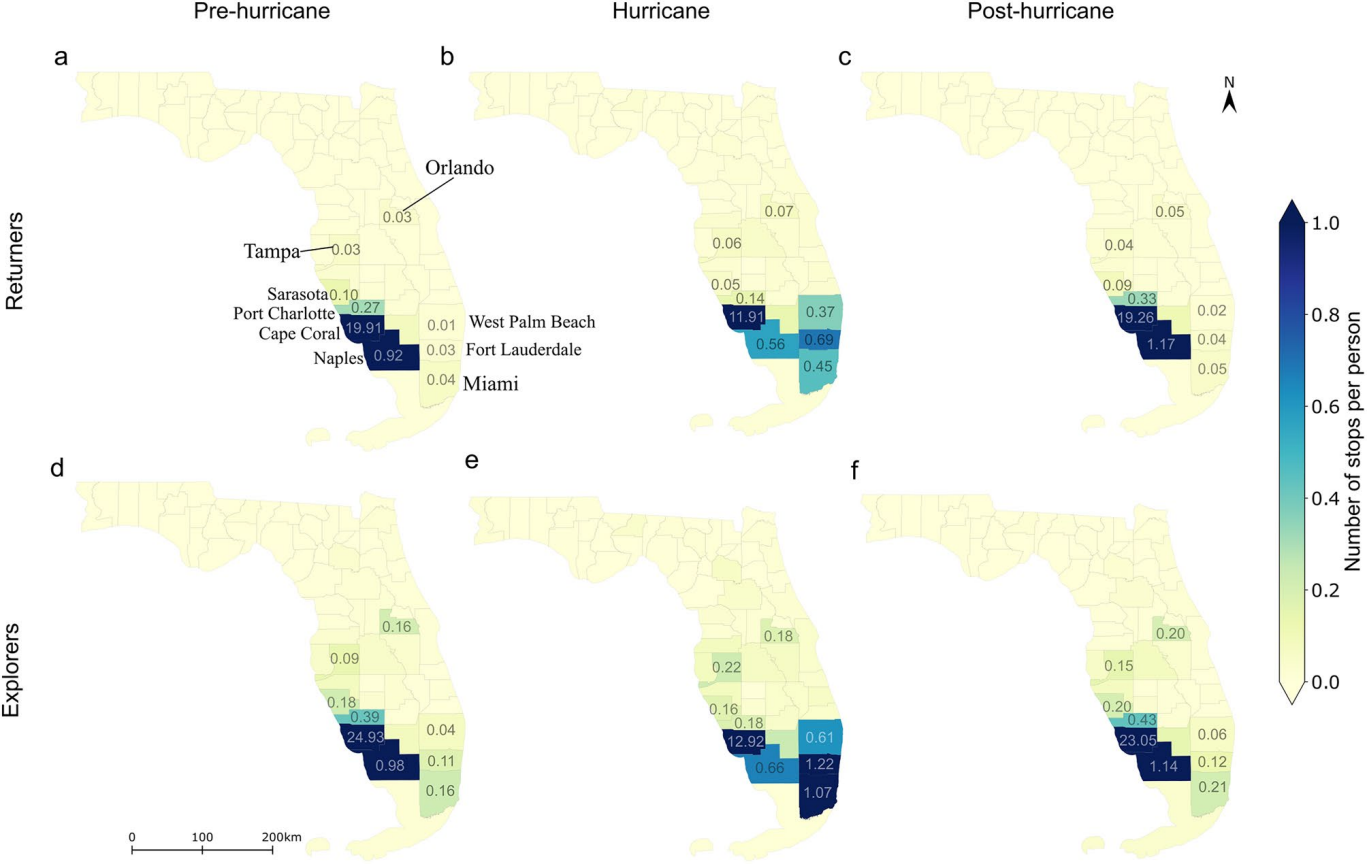
Spatial distribution of activity stops for returners (**a**–**c**) and explorers (**d**–**f**) across the three periods. The darker the color the greater the number of stops per person [Figure created using Geopandas 0.12.2: https://geopandas.org and Matplotlib 3.6.2: https://matplotlib.org].

Regarding spatial mobility patterns, Fig. 7 shows that during the hurricane period, activity stops from both groups gravitated toward shared destinations, primarily focusing on several major cities in proximity, such as Naples, West Palm Beach, Fort Lauderdale, and Miami. While some stops from both groups also appeared northward in Tampa and Orlando, these northbound stops were significantly less frequent compared to stops directed south and east to avoid the hurricane’s path. While some similarities exist between the two groups during the hurricane period, a more granular comparison is facilitated through a daily analysis. The findings are depicted in Supplementary Fig. 9. It is observed that on September 27th, one day before the hurricane’s landfall, the predominant distinctions in the spatial distribution of stops between the two groups were confined to Lee County. However, on the day of landfall, September 28th, more pronounced differences in the spatial distribution of stops emerged, particularly in Miami, indicating a greater tendency for explorers to seek shelter in that area. On September 29th, as the hurricane departed from Florida, the differences in the spatial distribution of stops within Lee County between the two groups started to recover. By September 30th, spatial variations in stops between the two groups were evident in Tampa, Orlando, and Collier County, aligning with pre-hurricane patterns, which indicate a restoration of movement patterns among Lee County residents close to a normal state.

Besides the hurricane phase, Fig. 7 and Supplementary Fig. 10 highlight significant distinctions in their movement behaviors during the pre-hurricane stage. Specifically, explorer displayed a significantly wider range of travel activities before the hurricane. The majority of their activities were concentrated in nearby cities, such as Port Charlotte and Naples, with some minor clusters in more distant large cities like Orlando, Tampa, Sarasota, Fort Lauderdale, and Miami. In contrast, most returner concentrated their activities in immediately nearby cities, including Port Charlotte and Naples. Furthermore, as depicted in Supplementary Fig. 11 and Supplementary Fig. 12, explorers typically exhibited longer durations at activity locations compared to returners across the three periods. Overall, while the spatial patterns of travel destinations differed significantly during non-hurricane periods, there was a general alignment in the spatial distribution of stops during hurricane, albeit with some localized disparities.

## Discussion

Utilizing extensive mobile phone location data from Lee County residents during Hurricane Ian, this study underscores the disruptive yet resilient nature of human movement patterns during hurricane conditions. The fitting and maximum likelihood estimation results reveal the persistent adherence of human movement to a truncated power-law distribution even under the influence of Hurricane Ian, reinforcing the predictability of human behavior during disasters. Although there are slight variations in the parameters of the fitted function before and after the hurricane, the overall movement pattern remains perturbed only to a limited extent. An intriguing observation is the shift in the probability distribution of the radius of gyration around 70 km, suggesting a potential threshold for long-distance travel choices, possibly from an increase in the number of locations visited and an increase in long-distance travel.

The dichotomy between returners and explorers becomes more pronounced during the hurricane than in normal circumstances. This is evident in the emergence of two distinct peaks in the distribution of $S_{k}$ at $S_{k} = 0$ (explorers) and $S_{k} = 1$ (returners). This pattern is particularly noticeable in the hurricane stage across various values of *k*, with emphasis on *k* = 3. It signifies that both groups exhibited their most distinct movement patterns, characterized by recurrent visits, in response to the hurricane. Furthermore, existing research indicates that explorers transition to become returners as *k* increases^16^, and this shift is particularly noticeable during hurricane events. Upon observing Fig. 4, it becomes evident that the prevalence of recurrent movements in overall mobility increases rapidly as *k* transitions from 2 to 3, corresponding to the swift shift in the percentage share of explorers and returners when the value of *k* changes from 2 to 3 in Supplementary Fig. 5. These observations suggest that the hurricane amplifies people’s inclination to explore new locations, potentially driven by evacuations or preparatory activities undertaken in response to hurricanes.

In the pre-hurricane stage, returners favored short-distance trips, contrasting with explorers who leaned towards longer journeys. This pattern persisted during the hurricane, but with a notable difference: both groups demonstrated a significant increase in the number of long-distance trips, which could be associated with evacuation behaviors. Moreover, although there were no statistically significant differences in the amount of time spent away from home between the two groups under normal conditions, during the hurricane, there was a clear polarization in their non-home dwelling times, with the proportions of staying at home versus continuous absence reaching their peaks.

In comparison to returners, explorers exhibited a higher tendency to travel during the hurricane. This inclination can be attributed to explorers’ natural inclination for discovering new locations, making them more predisposed to travel in the face of disasters. Nevertheless, among activity stops from both groups, there is a shared spatial preference, with Miami and Fort Lauderdale ranking as the most popular destinations, followed by Naples and West Palm Beach, aligning with previous research highlighting a preference for larger cities during evacuations^46^. Despite similarities in spatial preferences, the daily variation analysis reveals that, compared to returners, explorers had a greater propensity to stay in Miami, both in terms of location and duration of stay. This reflects their exploratory tendencies on one hand, and on the other hand, it indicates their susceptibility to being impacted. However, the common spatial preference in activity stops between the two groups could be influenced by Hurricane Ian’s path. As the hurricane gradually moved northeast, the direction of Miami became more favorable for travel compared to northward routes.

Analyzing the movement patterns of returners and explorers during emergency scenarios, like hurricanes, provides crucial insights for effective emergency management and rescue operations. To begin, it is imperative to acknowledge the unique mobility traits exhibited by returners and explorers. Subsequently, the development of evacuation plans should take into account the distinct preferences and behaviors of both groups. Secondly, recognizing the heightened propensity of explorers to travel during emergencies is crucial. Offering additional support and information for explorers, who are more inclined to explore unfamiliar areas, becomes imperative. While facilitating their need for exploration, it is essential to ensure safety measures are in place. Decision-makers might consider implementing real-time monitoring systems and timely alerts tailored to the tendencies of explorers. Providing information on safe routes, shelter locations, and essential services can guide their movements effectively. Thirdly, decision-makers should take into account the divergent non-home dwelling times during the hurricane and formulate communication strategies that cater to the distinct needs of individuals who remain homebound and those consistently outside. Customizing messages to promote preparedness, safety measures, and resource availability for both scenarios is essential. Fourthly, recognizing the identified shared spatial preferences for travel during the hurricane offers an opportunity for decision-makers to strategically allocate resources in line with popular travel destinations like Miami, Fort Lauderdale, Naples, and West Palm Beach. Collaboration with law enforcement to optimize routes based on observed patterns can enhance overall efficiency. By incorporating these policy implications into emergency management strategies, authorities can enhance the effectiveness of evacuation plans, resource allocation, and communication efforts, ultimately improving the overall resilience of communities during disasters.

This study is subject to certain limitations. The classification of returners and explorers can be influenced by the duration of observation. While the chosen four-day period may not be the optimal time scale for classification, the study’s natural constraint is attributed to the short-term nature of most hurricane events. Future research should explore the effects of disasters with different durations on the classification of returners and explorers, quantifying the impact of time scales on the analysis. Additionally, the high-resolution mobile phone location data holds the potential to extend the present county-analysis of evacuation behavior to a fine-grained level, such as census tracts. Conducting a more detailed intra-urban analysis would significantly enhance the accuracy and effectiveness of disaster relief operations, which is a focal point for our future work. Furthermore, while the dataset employed in this study boasts high spatiotemporal resolution, it is susceptible to significant reductions in data volume during hurricane periods due to various disturbances, such as power outages and signal loss. This is an inherent limitation of mobile location data, which could potentially be mitigated in future research by expanding the scope of the study area to include counties along the entire hurricane path, thereby mitigating the impact of disasters on data volume. Additionally, despite the data’s high positioning accuracy, it may experience some degree of signal drift or anomalous positioning information. To improve this issue, one might consider matching positioning data to road networks to further ensure good quality of the data. It is also noteworthy that this study focuses solely on the presence of returners and explorers in disaster scenarios and their spatiotemporal mobility patterns in response to hurricane impacts, without delving into the underlying factors driving these changes, such as the socioeconomic status composition of both groups, and the relationship between mobility pattern shifts and hurricane intensity. Future research will prioritize these aspects. Lastly, a future comparative study is necessary to determine if the identified mobility patterns are applicable to different study locations and disaster scenarios.

## Methods

### Date and preprocessing

The mobility dataset for this study comprises privacy-preserving mobile phone GPS data sourced from the location intelligence firm Cuebiq. The data are collected from various smartphone applications, through which anonymous users opt-in to provide access to their location data anonymously through a process compliant with the California Consumer Privacy Act. In addition to anonymizing the data, Cuebiq excludes visits from Sensitive Points of Interest, and obfuscates home locations at the Census Block Group level in order to preserve privacy. Each entry in the data includes: a de-identified user ID, latitude, longitude, timestamp, and positioning accuracy. Cuebiq also offers supplementary tables derived from the data, such as those detailing users’ activity stops. The data are already curated by Cuebiq; they apply machine learning algorithms to detect and delete outliers based on multiple criteria including positioning accuracy, inferred speed, and geography. Compared with mobile phone CDR in which the user’s location is recorded only when the connection to cellular network is established, e.g., making/receiving calls, this dataset provides much finer grained location traces. Several recent studies have demonstrated the high validity of Cuebiq’s mobile phone data^47,48^.

To elaborate further, activity stops are derived from a stop-detection algorithm^49^ that enables us to identify which sequence of points constitutes a single stay of a user, subsequently aggregating them into a singular entity characterized by latitude, longitude, and duration (referred to as “dwell time”). Essentially, this algorithm is a spatiotemporal clustering model designed to automatically extract stops from a single trajectory^49^. It firstly calculates the Core Distance, which represents the spatial closeness of a trajectory point to its temporal neighbors. The Core Distance is computed for every point of the trajectory. Then, consecutive points with a Core Distance smaller than Epsilon are aggregated, where Epsilon is a parameter that the data provider tuned while implementing the algorithm. To ensure accuracy, the Epsilon parameter has been made adaptive to account for the lack of precision of certain points. In our study, the aggregation threshold for time is set at two minutes; hence, a single stop in our research may encompass numerous original stay points. This approach allows for a more accurate reflection of genuine stay information while minimizing the inclusion of transient stops, such as those caused by traffic. Additionally, data entries documenting dwell times exceeding 24 h per day are considered outliers and are thus removed. Furthermore, for the analysis across different periods, stops pertaining to all individuals with at least one record spanning four consecutive days in each of the three examined periods were filtered, Supplementary Table 5 presents the final accuracy details of the data.

### Fundamental mobility metrics

To analyze variations in human movement across different study periods, this study first evaluates individuals’ displacement and radius of gyration, which serves as the basis for the subsequent identification of returners and explorers. The radius of gyration and displacement are key metrics in assessing human mobility behavior. The radius of gyration delineates the spatial extent of an individual’s movement, and displacement quantifies travel distance, serving as a proxy for willingness to travel. These metrics play a crucial role in gauging the resilience or disruption of mobility patterns during emergencies, such as hurricanes. Three prevalent distribution functions are employed to model these movement patterns and compare their fits. Using the identified stop table provided by Cuebiq, the displacement for a user will be measured by the great-circle distance between two consecutive stops, following many existing studies^28,29^. The great-circle distance can be calculated using the following Haversine formula^50^:

1 $$\begin{array}{*{20}c} {D = 2r \times sin^{ - 1} \left( {\sqrt {sin^{2} \left( {\frac{{\phi_{m} - \phi_{n} }}{2}} \right) + cos\phi_{m} cos\phi_{n} sin^{2} \left( {\frac{{\varphi_{m} - \varphi_{n} }}{2}} \right)} } \right)} \\ \end{array}$$

where *r* is the radius of the Earth, and $\varphi$ and $\phi$ denote the longitude and latitude of the stop location, respectively. The radius of gyration measures the typical distance of traveled by a user from the center of mass of their trajectory^20^:

2 $$\begin{array}{*{20}c} {r_{g} = \sqrt {\frac{1}{N}\mathop \sum \limits_{i \in L} n_{i} \left[ {2r \times sin^{ - 1} \left( {\sqrt {sin^{2} \left( {\frac{{\phi_{i} - \phi_{c} }}{2}} \right) + cos\phi_{c} cos\phi_{i} sin^{2} \left( {\frac{{\varphi_{i} - \varphi_{c} }}{2}} \right)} } \right)} \right]^{2} } } \\ \end{array}$$

where *L* represents the set of meaningful stop locations, $\varphi_{i}$ and $\phi_{i}$ represent the longitude and latitude of location $i$, $\varphi_{c}$ and $\phi_{c}$ are the longitude and latitude of the center of mass of the individual’s moving range, $n_{i}$ is the frequency of visits to location $i$, and *N* is the total number of stop locations.

Three distribution functions are used to fit the displacements and radius of gyration: the exponential distribution, ortho-logarithmic distribution, and truncated power-law distribution:

3 $$\begin{array}{*{20}c} {P_{1} (x) \propto e^{ - \lambda x} } \\ \end{array}$$

4 $$\begin{array}{*{20}c} {P_{2} (x) \propto \frac{1}{x}exp\left[ { - \frac{{\left( {\ln x - \mu } \right)^{2} }}{{2\sigma^{2} }}} \right]} \\ \end{array}$$

5 $$\begin{array}{*{20}c} {P_{3} (x) \propto x^{ - \alpha } e^{ - \lambda x} } \\ \end{array}$$

where *α* is the scaling parameter, *λ* is the exponential cutoff value, and $\mu$ and $\sigma$ are the mean and standard deviation, respectively. The maximum likelihood estimation^51^ is employed to rigorously assess the fit quality of these distributions. The fitting and comparison are conducted using the powerlaw Python package^52^.

This study also computes the real entropy, another fundamental metric to evaluate the predictability of mobility patterns. This metric offers the advantage of encapsulating not only the frequency of individual visits but also the sequence of visited nodes and the duration spent at each location, thereby capturing the complex spatiotemporal order inherent in an individual’s mobility patterns^53^. It can be formulated as follows^54^:

6 $$\begin{array}{*{20}c} {E\left( u \right) = - \mathop \sum \limits_{{T^{\prime}_{u} }} P\left( {T^{\prime}_{u} } \right)log_{2} \left[ {P\left( {T_{u}^{i} } \right)} \right]} \\ \end{array}$$

where $P( {T^{\prime}_{u} })$ is the probability of finding a particular time-ordered subsequence $T^{\prime}_{u}$ in the trajectory $T_{u}$. The higher the value the higher the unpredictability of the individual.

### Classification of returners and explorers

To differentiate between returners and explorers, this study employs the *k*-radius of gyration, which serves to characterize how the *k* most frequently visited locations by individuals influence their characteristic distance of movement^16^:

7 $$\begin{array}{*{20}c} {r_{g}^{( k )} = \sqrt {\frac{1}{{N_{k} }}\mathop \sum \limits_{i = 1}^{k} n_{i} \left[ {2r \times sin^{ - 1} \left( {\sqrt {sin^{2} \left( {\frac{{\phi_{i} - \phi_{c}^{( k )} }}{2}} \right) + cos\phi_{c}^{( k )} cos\phi_{i} sin^{2} \left( {\frac{{\varphi_{i} - \varphi_{c}^{( k )} }}{2}} \right)} } \right)} \right]^{2} } } \\ \end{array}$$

where $\varphi_{c}^{( k )}$ and $\phi_{c}^{( k )}$ represent the longitude and latitude of the center of mass calculated based on the top *k* frequently visited locations of the individual, and $N_{k}$ is the sum of the weights assigned to the top *k* most frequently visited locations. Then returners and explorers can be classified by comparing $r_{g}^{( k )}$ and $r_{g} /2$ of individuals:

8 $$\begin{array}{*{20}c} {\left\{ {\begin{array}{*{20}c} {k - returner:\;\; r_{g}^{( k )} > r_{g} /2} \\ {k - explorer:\;\; r_{g}^{( k )} < r_{g} /2} \\ \end{array} } \right.} \\ \end{array}$$

Furthermore, to highlight the impact of an individual’s top *k* frequently visited locations on their overall mobility, this study examines the metric $S_{k} = r_{g}^{( k )} /r_{g}$. A higher value of $S_{k}$ indicates a stronger inclination of an individual to predominantly visit those $k$ locations.

### Analysis of spatiotemporal mobility patterns

To analyze the spatiotemporal mobility patterns across the three specified time periods for both returners and explorers, this study identifies all individuals from each mobility group who have traveled on a given day. It then calculates the maximum distance from home and the duration of stay away from home for each mobility group during each period. Furthermore, it examines the spatial distribution of activity stops and the length of stay across Florida for both groups. When examining changes in mobility patterns among returners and explorers, the maximum distance from home offers a valuable insight into individual mobility dynamics compared to displacement. This distinction arises from the typically consistent daily travel distance from home under normal circumstances. Any significant deviation from this norm may signify abnormal mobility responses triggered by a hurricane. Consequently, various studies have employed distance from home as a pivotal indicator for assessing human mobility patterns^19,55^. The duration of stay away from home metric describes the stability of time spent at home versus away, allowing for straightforward interpretation of changes in mobility behavior. By focusing on (non)home time, researchers can discern disruptions to individuals’ typical routines, which often signify shifts in their overall mobility patterns^56,57^. Moreover, metrics associated with home provide a distinct advantage by incorporating a stable reference point—home itself—into the analysis. This inclusion effectively filters out random or inconsequential movements or stays, thereby enhancing the reliability of assessing changes in human mobility patterns.

### Supplementary Information

Supplementary Information.

## Data Availability

The mobile phone data that support the findings of this study are available from Cuebiq Inc. but restrictions apply to the availability of these data, which were used under license for the current study, and so are not publicly available. However, the data may be accessed upon reasonable request to both the corresponding author and Cuebiq Inc.
