# Supplementary material for: Returners and explorers dichotomy in the face of natural hazards
Source: Sci Rep. 2024 Jun 8;14:13184. doi: 10.1038/s41598-024-64087-4 (PMC11162431; doi:10.1038/s41598-024-64087-4)
Supplement: Supplementary file 1 — Supplementary Information. [file 41598_2024_64087_MOESM1_ESM.docx]

***Supplementary Information for:***

**Returners and Explorers Dichotomy in the Face of Natural Hazards**

Zeyu He^1^, Yujie Hu^1,*^, Leo L. Duan^2^, George Michailidis^3^

^1^Department of Geography, University of Florida, Gainesville, FL 32611

^2^Department of Statistics, University of Florida, Gainesville, FL 32611

^3^Department of Statistics and Data Science, University of California Los Angeles, Los Angeles, CA 90095

*Corresponding author: Yujie Hu (Email: yujiehu@ufl.edu)

**1. Supplementary Figures**
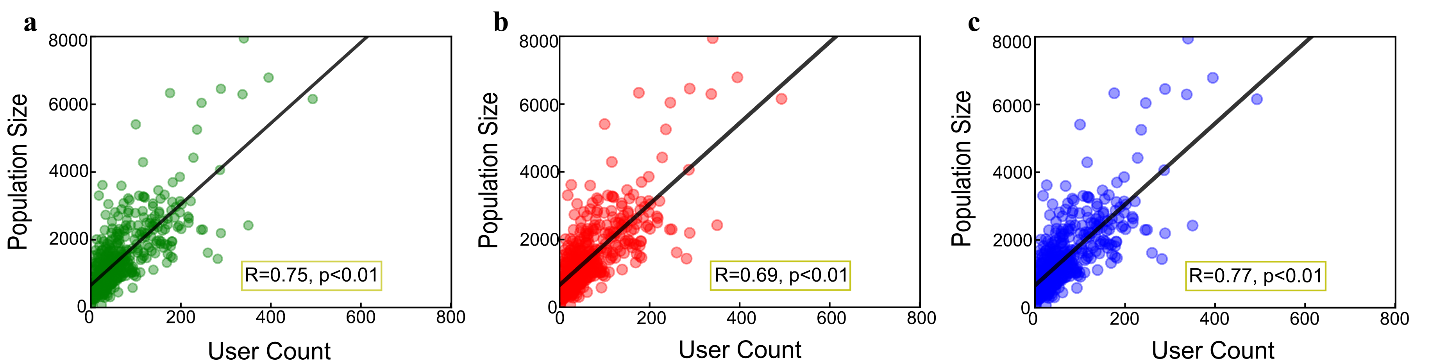


**Supplementary Figure 1.** Correlation between population size and user count at the census block group level for pre-hurricane (a), hurricane (b), and post-hurricane (c) periods. This is measured by comparing the population inferred from the mobility data with the census population data from the 2014-2018 American Community Survey (ACS) at the census block group level.


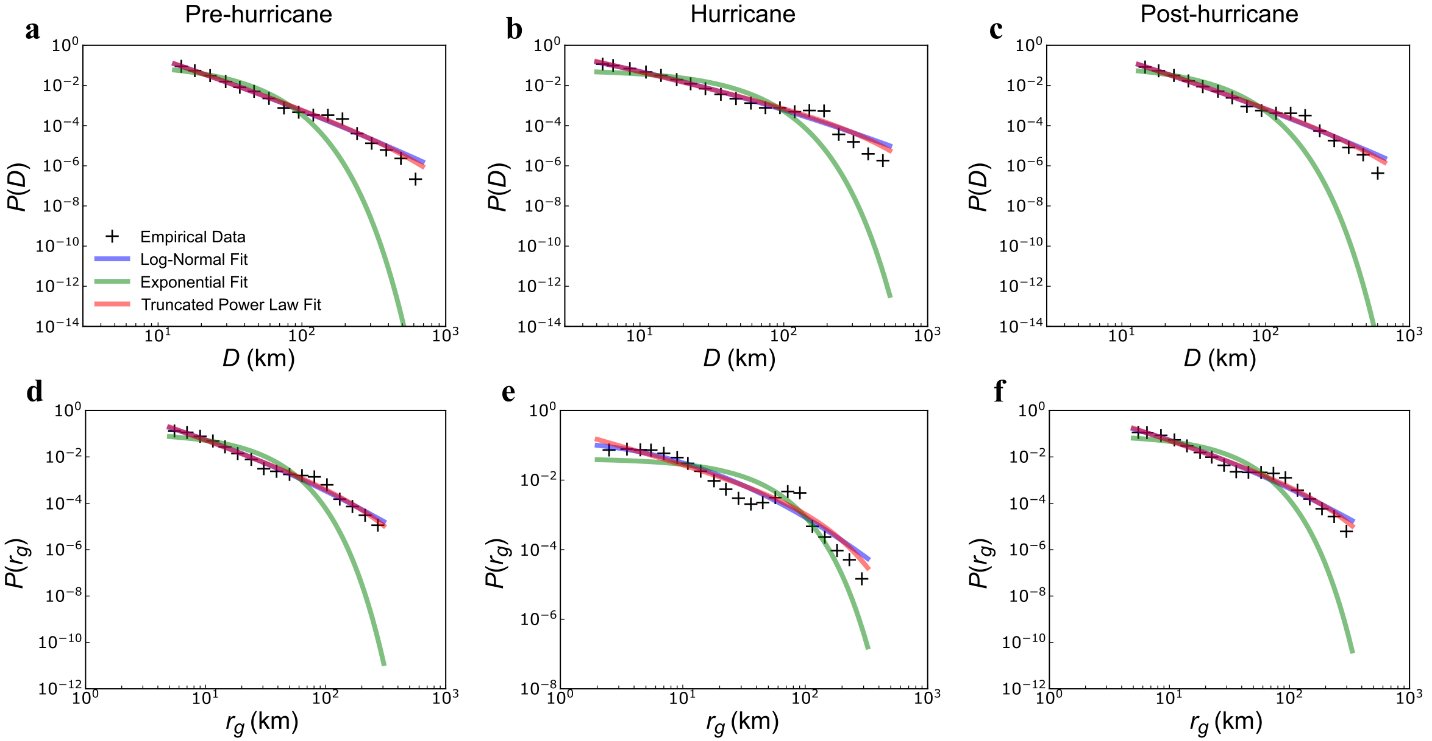


**Supplementary Figure 2.** Fitting results for the three stages. Parts (a-c) provide detailed displacement fitting results, while parts (d-f) show radius of gyration fitting results. Black crosses denote the probability distribution of the raw data, while the solid red line illustrates the truncated power law distribution fit. The exponential and lognormal distribution fits are shown by solid green and blue lines, respectively.


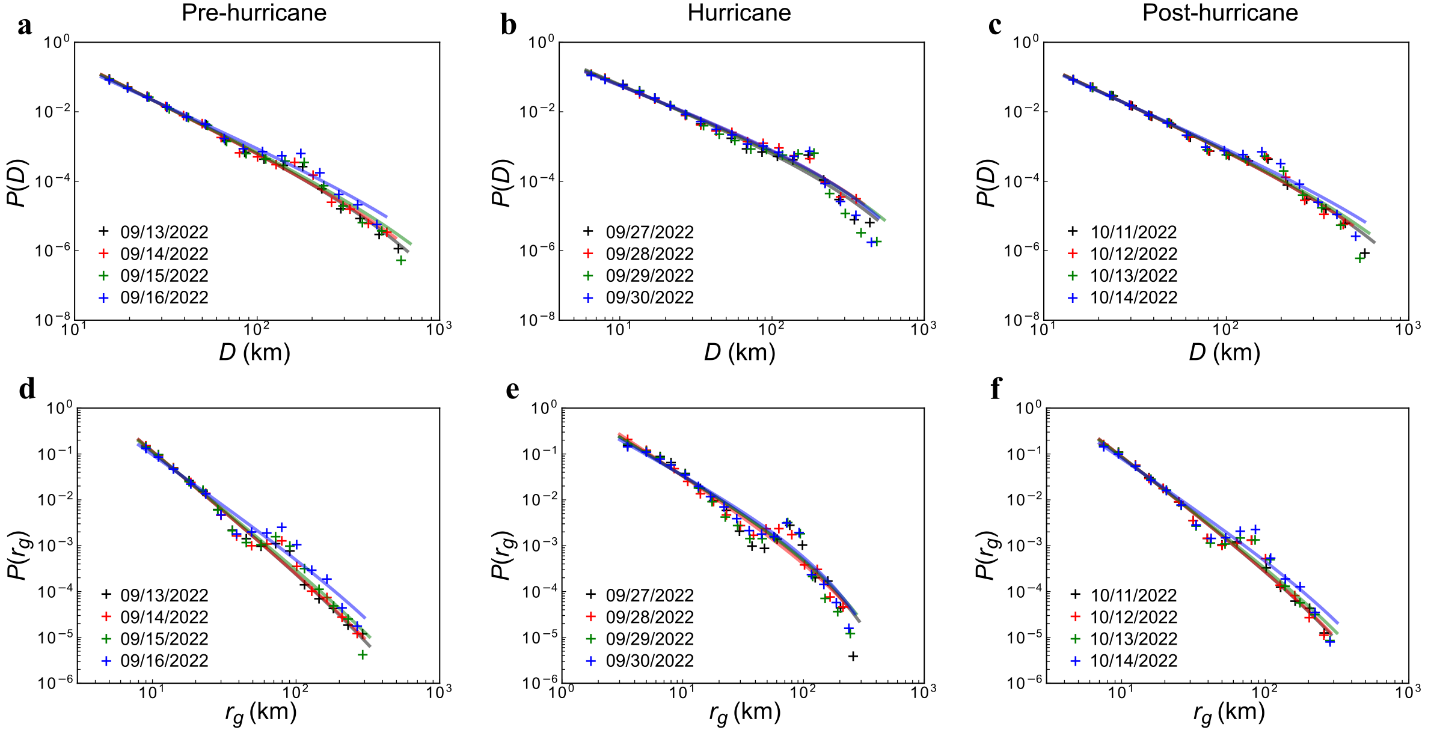


**Supplementary Figure 3.** Daily fitting results. Subplots a-c provide detailed displacement fitting results, while subplots d-f show radius of gyration fitting results. Crosses depict individual data points, while the various colored solid lines signify the truncated power-law fits.


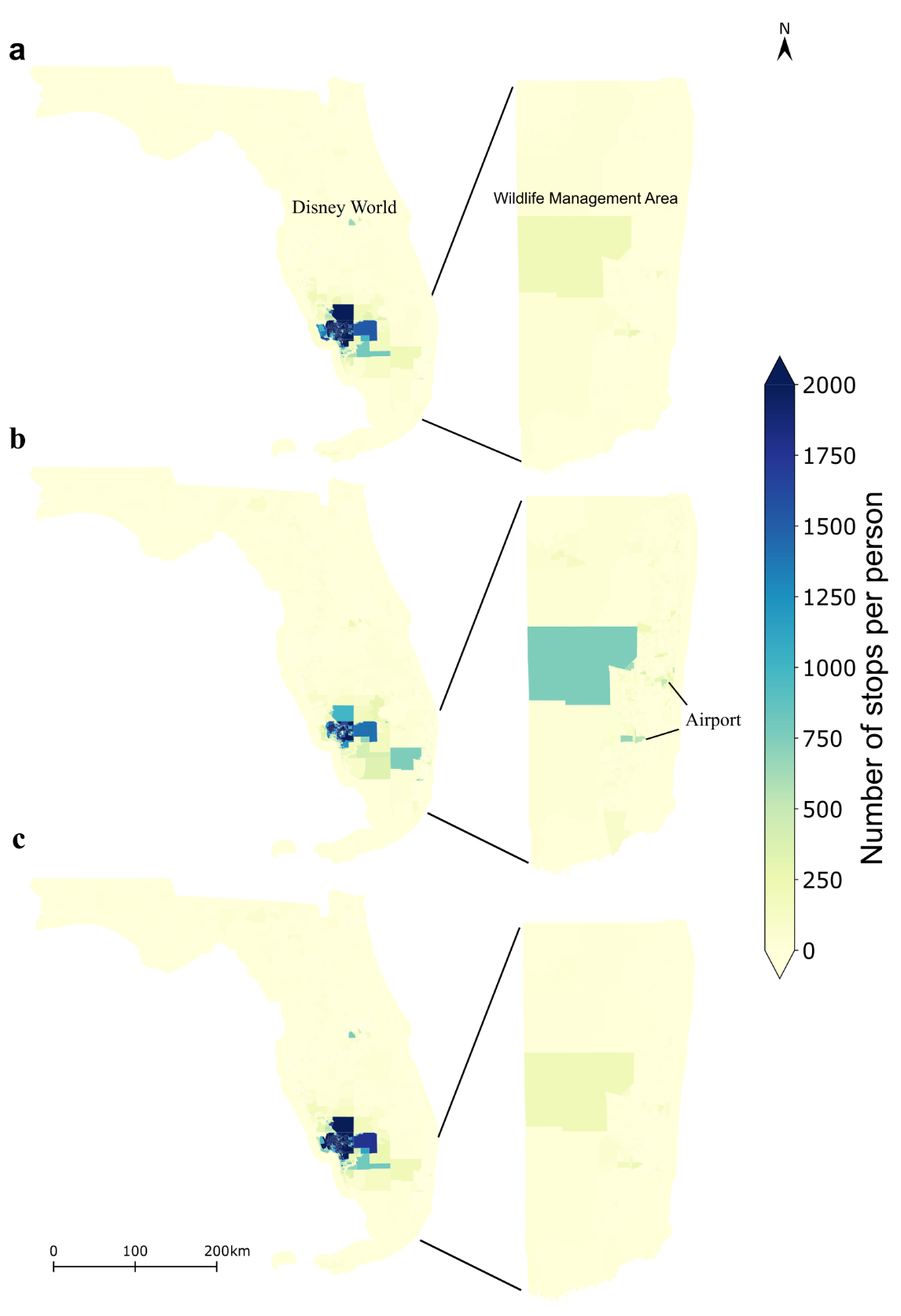


**Supplementary Figure 4.** Distribution of activity stops by Lee County residents in the pre-hurricane (a), hurricane (b), and post-hurricane periods.


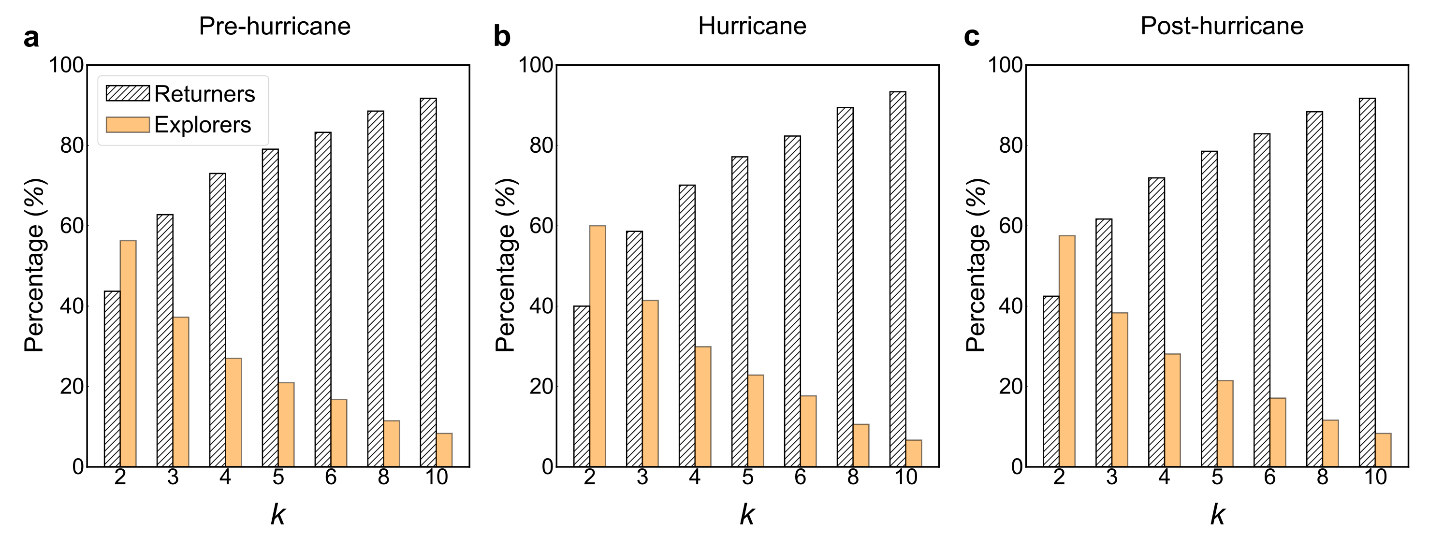


**Supplementary Figure 5.** Percentage of *k*-returners and *k*-explorers across the three periods.


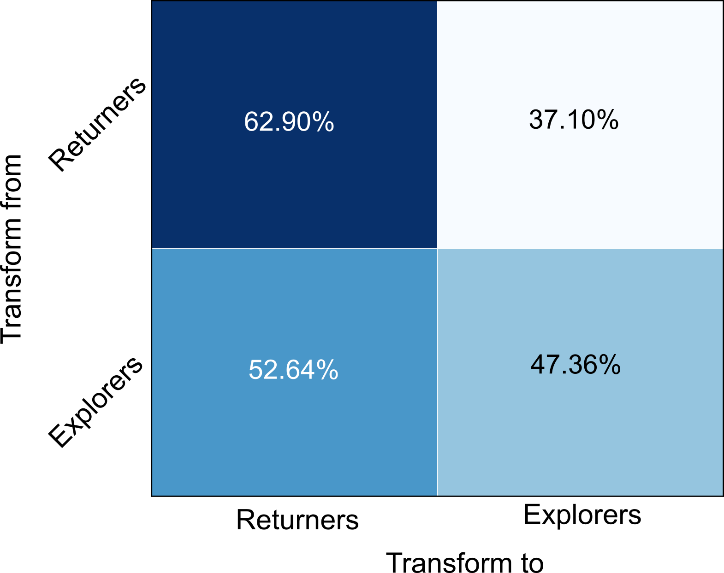


**Supplementary Figure 6.** Transformations between returners and explorers during the hurricane.


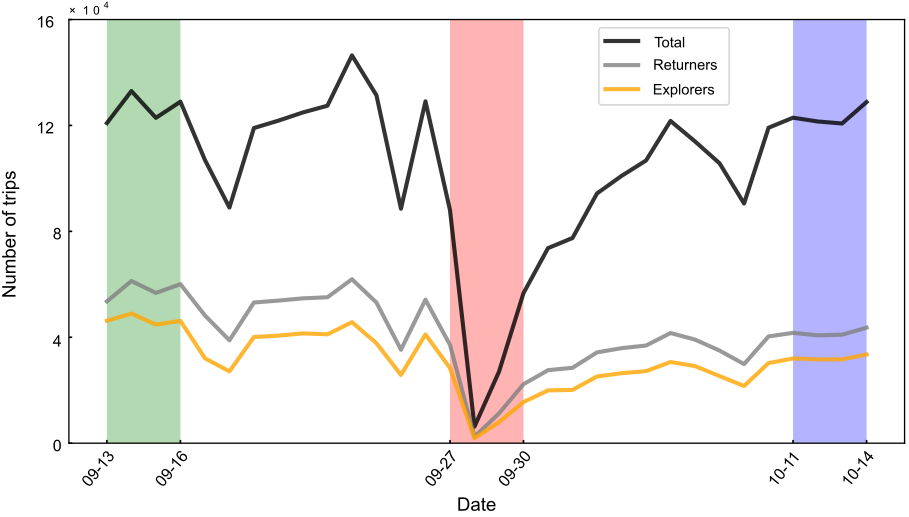


**Supplementary Figure 7.** Daily number of trips for returners and explorers between 09/13 and 10/14. The three vertical bands correspond to the three study periods.


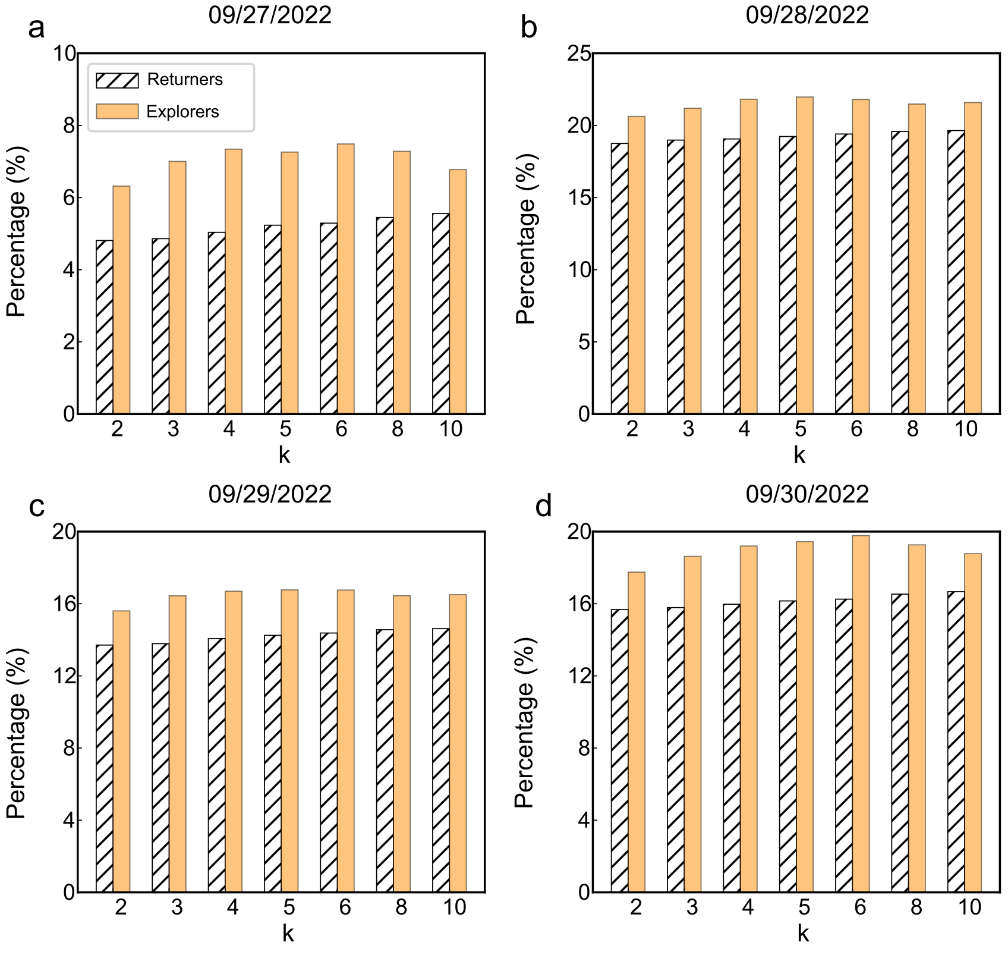


**Supplementary Figure 8.** Comparison of travel percentages between returners and explorers during the hurricane period.


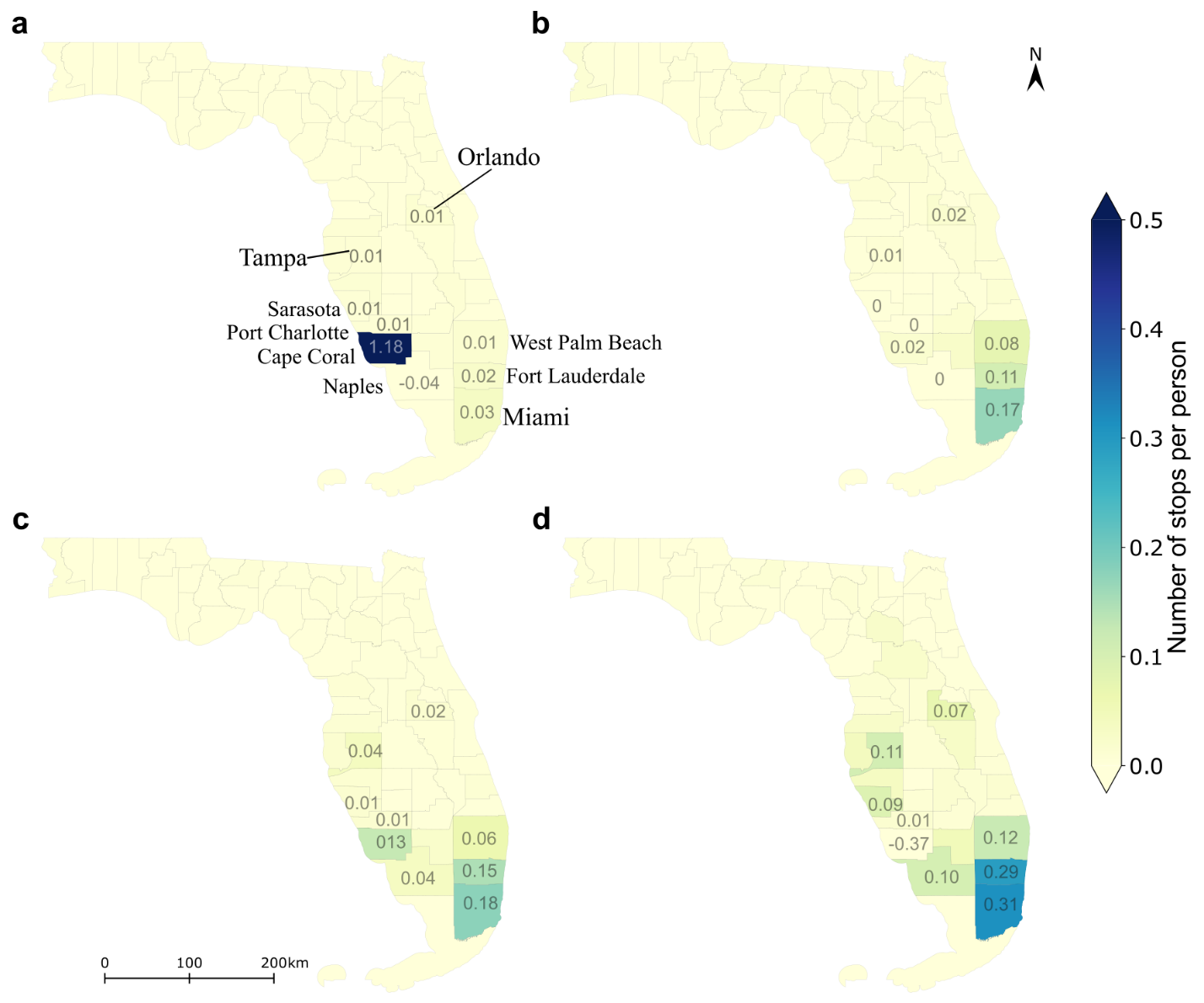


**Supplementary Figure 9.** Daily comparison maps illustrating differences in activity stops’ spatial distribution between returners and explorers on September 27 (a), 28 (b), 29 (c), and 30 (d). The discrepancy values are obtained by subtracting the values of returners from the values of explorers.


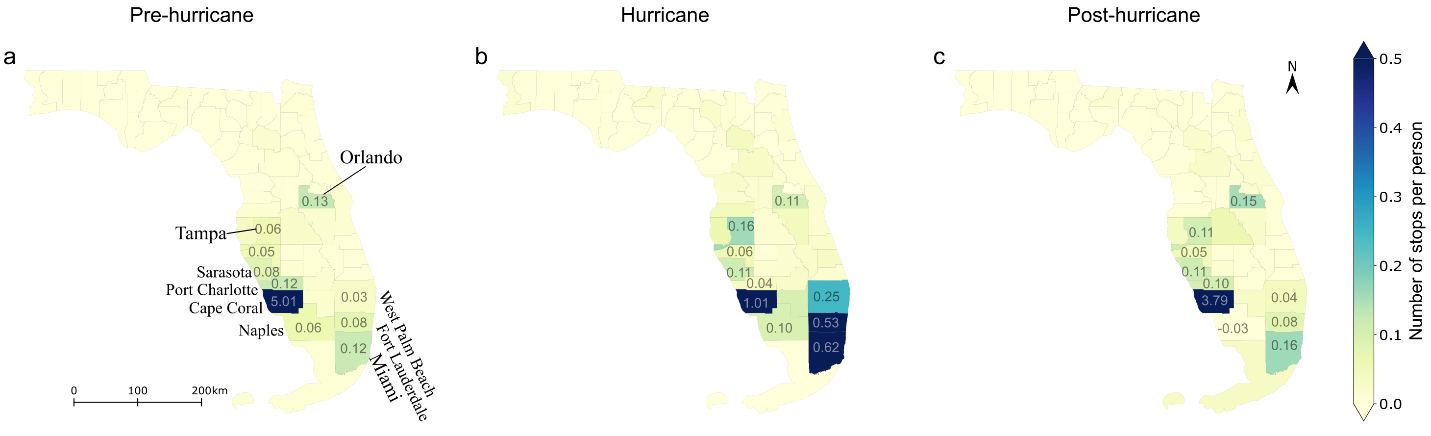


**Supplementary Figure 10.** Differences in the spatial distribution of activity stops between returners and explorers across the three periods. The values for the number of stops per person are obtained by subtracting the values of returners from the values of explorers in Fig. 7 in the main text.


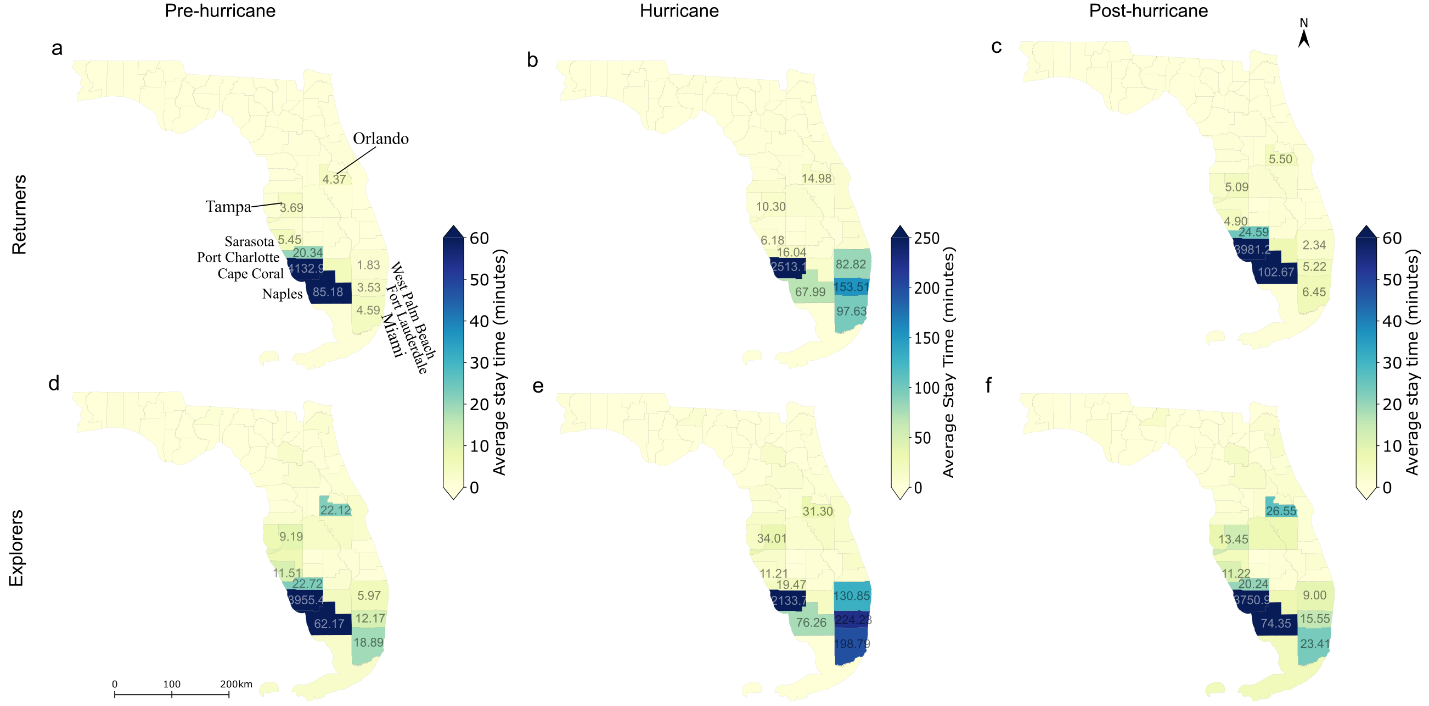


**Supplementary Figure 11.** Average duration of stay at stop locations for returners and explorers.


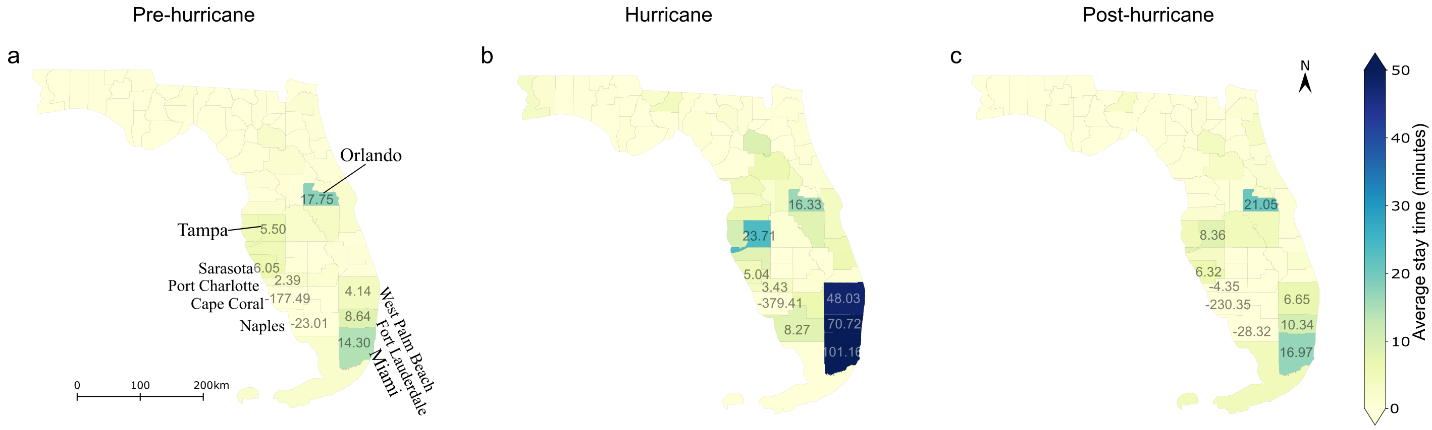


**Supplementary Figure 12.** Difference in the spatial distribution of average stay time at stop locations between returners and explorers. The average stay time values are obtained by subtracting the values of returners from the values of explorers.

**2. Supplementary Tables**

**Supplementary Table 1.** Results of maximum likelihood estimation.

| Study period | Mobility metric | Scaling parameter^1^ | $X\_min$^2^ | Exponential distribution^3^ | Lognormal distribution^4^ |
| --- | --- | --- | --- | --- | --- |
| Pre-hurricane | D | 2.493, 0.003 | 13 | 68.50 | 5.71 |
|  | $r_{g}$ | 1.845, 0.007 | 5 | 41.43 | 5.22 |
| Hurricane | D | 1.676, 0.004 | 5 | 95.77 | 13.98 |
|  | $r_{g}$ | 1.000, 0.011 | 2 | 32.68 | 12.00 |
| Post-hurricane | D | 2.381, 0.003 | 13 | 72.67 | 9.40 |
|  | $r_{g}$ | 1.709, 0.008 | 5 | 43.38 | 8.82 |

Note: ^1^The first value indicates the value of $\alpha$, while the second denotes the value of $\lambda$. ^2^$X\_min$ represents the minimum distance used for fitting the truncated power-law distribution. ^3^Positive values indicate a better fit to the truncated power-law distribution, whereas negative values indicate a better fit to the exponential distribution. ^4^Positive values indicate a better fit to the truncated power-law distribution, whereas negative values indicate a better fit to the lognormal distribution. All results are statistically significant at the 0.001 level.

**Supplementary Table 2.** Fitting parameters across *k* values of 2, 3, 4, and 8.

|  | Pre-hurricane | | Hurricane | | Post-hurricane | |
| --- | --- | --- | --- | --- | --- | --- |
|  | $\alpha$ | $\lambda$ | $\alpha$ | $\lambda$ | $\alpha$ | $\lambda$ |
| $r_{g}^{(2)}$ | 2.616 | 0.0004 | 1.656 | 0.0082 | 2.475 | 0.0019 |
| $r_{g}^{(3)}$ | 2.424 | 0.0038 | 1.448 | 0.0100 | 2..260 | 0.0056 |
| $r_{g}^{(4)}$ | 2.303 | 0.0058 | 1.306 | 0.0109 | 2.127 | 0.0076 |
| $r_{g}^{(8)}$ | 2.096 | 0.0078 | 1.052 | 0.0119 | 1.945 | 0.0085 |
| $r_{g}$ | 1.845 | 0.0074 | 1.000 | 0.0106 | 1.709 | 0.0078 |

**Supplementary Table 3.** Significance test for travel percentage, maximum distance from home, and non-home dwelling time between returners and explorers across the three periods.

|  | Travel percentage | | | | MDH and NDT | | | | | |
| --- | --- | --- | --- | --- | --- | --- | --- | --- | --- | --- |
|  | 09/27/2022 | 09/28/2022 | 09/29/2022 | 09/30/2022 | Pre-hurricane | | Hurricane | | Post-hurricane | |
|  |  |  |  |  | MDH | NDT | MDH | NDT | MDH | NDT |
| Mann-Whitney U Test results – statistical significance | ** | ** | ** | ** | ** | NS | * | NS | ** | NS |

Note: * means significant at the 0.05 level, ** significant at 0.01 level, MDH maximum distance from home, NDT non-home dwelling time, and NS non-significant.

**Supplementary Table 4.** Significance test for real entropy difference between returners and explorers across the three periods.

|  | Pre-hurricane | Hurricane | Post-hurricane |
| --- | --- | --- | --- |
| Mann-Whitney U Test results – statistical significance | *** | *** | *** |

Note: *** means significant at the 0.001 level.

**Supplementary Table 5.** Summary of the movement data across the three periods.

|  | Parameter | Pre-hurricane | Hurricane | Post-hurricane |
| --- | --- | --- | --- | --- |
| AD (meter) | Max | 192.9 | 199.0 | 232.7 |
|  | Min | 0 | 0 | 0 |
|  | Average | 8.4 | 9.2 | 8.6 |
|  | Median | 7.0 | 8.2 | 7.4 |
| AA (meter) | Max | 149.6 | 149.7 | 149.6 |
|  | Min | 1.0 | 1.0 | 1.0 |
|  | Average | 15.9 | 16.2 | 15.4 |
|  | Median | 12.7 | 12.0 | 12.1 |
| SA (meter) | Max | 104.3 | 100.1 | 101.1 |
|  | Min | 0 | 0 | 0 |
|  | Average | 7.4 | 8.8 | 7.8 |
|  | Median | 5.2 | 5.8 | 5.3 |
| NL (meter) | Max | 172.0 | 88.0 | 188.0 |
|  | Min | 4.0 | 4.0 | 4.0 |
|  | Average | 23.8 | 16.5 | 23.3 |
|  | Median | 21.0 | 15.0 | 20.0 |

Note: AD (Average Distance) is the average distance between barycenter (mean center of all points clustered in a stop) and original location points that have been clustered in a stop; AA (Average Accuracy) is the average accuracy of original location points; SA (Standard Deviation of Accuracy) is the standard deviation of the accuracy distribution of original points; and NL (Number of Locations) is the number of locations visited by an individual.
